# Attitudes towards Ambiguity in Japanese Healthy Volunteers

**DOI:** 10.1007/s12144-017-9569-9

**Published:** 2017-03-10

**Authors:** Hiroyuki Enoki, Munenaga Koda, Satona Saito, Sayako Nishimura, Tsuyoshi Kondo

**Affiliations:** 1 Department of Neuropsychiatry, Graduate School of Medicine, University of the Ryukyus, 207 Uehara, Nishihara, Okinawa, 903-0215 Japan; 2 Heiwa Hospital, Okinawa, Japan; 3 Kyoto University of Education, Kyoto, Japan

**Keywords:** Ambiguity tolerance–intolerance, Attitude, Uncertainty, AAQ, Personality, Factor analysis

## Abstract

Multi-dimensional structure of the Attitudes Towards Ambiguity Scale (ATAS: original Japanese version) and its relationship with the Acceptance and Action Questionnaire (AAQ) were investigated. We administered the ATAS and the Japanese version of the AAQ to 1019 Japanese healthy volunteers (513 females and 506 males; age range 18–78 years). Trial of exploratory factor analysis extracted four distinct clusters (Enjoyment; α = .83, Anxiety; α = .75, Exclusion; α = .75, and Noninterference; α = .65) from the ATAS item pool, suggestive of diversity in cognitive/ emotional/ behavioral responses to ambiguity. Confirmative factor analysis showed similar goodness in fit indices between the new four-factor model in the present study and the original five-factor model in our previous study (Nishimura 2007). Considering interpretability by using large number of representative samples with general population in the present study, we adopted the four-factor model. The ATAS Anxiety subscale was negatively correlated with the AAQ willingness subscale (*r* = −.39, *p* < .001), while the ATAS Enjoyment subscale was positively correlated with the AAQ Action subscale (*r* = .40, *p* < .001). It is thus suggested that one who enjoys ambiguous situations can adopt two distinct attitudes: Excluding ambiguity from active resolution, or not interfering with ambiguity due to good tolerance of this experience, which can lead to positive and flexible commitments in life. In contrast, one who tends to be anxious about ambiguity may be characterized by exclusion-based attitudes due to intolerance of ambiguity, leading to lowered acceptance of their feelings and of the reality of circumstances. Cognitive/emotional attitudes towards ambiguity may affect acceptance of inner experience and active commitment to reality.

Frenkel-Brunswik (1949) originally proposed the concept of tolerance of ambiguity. She considered tolerance of ambiguity to be an aspect of personality and found low levels of this trait in authoritarian personalities. Subsequently, numerous researchers have investigated various aspects of this construct. Budner (1962) defined ambiguity as an unstructured and undifferentiated state that arises due to insufficient clues to clarify a situation and allow for its comprehension. He classified ambiguous situations into the following three types: 1) a completely unfamiliar situation without any clues (novelty), 2) a complicated situation with too many clues (complexity) and 3) a contradictory situation due to confounding clues (insolubility).

However, there have been criticisms regarding the tolerance of ambiguity concept, based on various aspects of the psychometric evidence (Herman et al. 2010). First, it remains controversial whether tolerance of ambiguity is truly related to individual personality traits (Budner 1962; MacDonald 1970; McLain 1993) or whether it merely reflects a situation-dependent/content-specific expression of psychological stress (Durrheim 1998). Second, although tolerance of ambiguity has been quantitatively operationalized as a unitary model, qualitative assessments of multi-dimensional aspects of attitudes towards ambiguity seem to represent a more realistic and attractive approach (Furnham and Marks 2013; Furnham and Ribchester 1995). Third, although tolerance of ambiguity has been investigated primarily with regard to negative responses to ambiguous stimuli such as threat, discomfort, and anxiety (Grenier et al. 2005), positive cognitive and emotional responses such as curiosity and attraction towards ambiguous situations can also occur, as suggested by Montuori (2005) and Zenasni and Lubart (2008), who found some aspects of attitudes towards ambiguity to be associated with creativity. Accordingly, the field should pursue a comprehensive understanding that includes both positive and negative aspects of attitudes toward ambiguity (McLain et al. 2015). It is important to analyze diversity in personal cognitive/affective/behavioral patterns regarding ambiguous situations, rather than simply assessing individual tolerance to ambiguous stimuli.

Durrheim and Foster (1997) regarded tolerance of ambiguity as a multidimensional attitude, which they discussed in mainly in the social context of political attitudes. Purely from a psychological point of view, dynamic aspects of personal attitudes towards ambiguity (such as multidimensional cognitive/emotional responses) would be more important to emphasize, rather than static aspects of public tolerance of ambiguity in social situations. However, very few studies have emphasized such an approach to date.

Recently, Nishimura (2007) developed the Attitudes Towards Ambiguity Scales (ATAS: the original version in Japanese), to represent a multi-dimensional model of assessing response to ambiguity. This scale, containing 26 items, was originally developed to assess attitudes towards ambiguity as personal traits, including one’s evaluation of ambiguous situations (cognitive/emotional responses) and orientation for decision-making (behavioral patterns), rather than to merely quantify tolerance of ambiguity. Factor analysis of the responses of 437 university students (Nishimura 2007) revealed a five-factor model of attitudes towards ambiguity, consisting of two positive attitudes (enjoyment and reception) and three negative attitudes (anxiety, control, and exclusion). Nishimura (2007) later found satisfactory reliability, validity, and reproducibility of this scale. The same author (Nishimura 2007) also reported some relationships between attitudes towards ambiguity and common psychological reactions such as depressive and obsessive tendencies, indicating moderate correlations between the anxiety subscales of the ATAS and the Zung Self-rating Depression Scale (Zung 1965), as well as the Maudsley Obsessional Compulsive Inventory (Hodgson and Rachman 1977).

Only one study (Tsuda 2015) conducted confirmatory factor analysis of the ATAS in female dominant and all young subjects (197 college students, proportion of females: 68.5%, mean age ± *SD*: 19.6 ± 0.7), based on the five-factor model proposed by Nishimura (2007). However, small and age−/gender-biased samples in the Tsuda’s study (Tsuda 2015) did not fulfill criteria for samples suitable for confirmatory factor analysis according to previous studies (Hu and Bentler 1999; Jackson 2003 and Kline 2011). Thereby, larger representative samples of general population with wider age range and even gender distribution will be requisite to conduct correct confirmatory factor analysis.

The original Japanese version of the ATAS remains the only version currently available and thus is suitable only for use with Japanese populations. However, using the ATAS as a probe for multidimensional attitudes towards ambiguity among the Japanese population is certainly a worthwhile endeavor, given a relative paucity of such studies even amongst English-speaking populations. However, Nishimura (2007) proposed the five-factor model of the ATAS on the basis of preliminary data from only a small sample of university students. Therefore, a re-assessment of this model using a larger sample across a wider age range is solely needed.

It is also of great interest for us to determine the relationship between the ATAS and other established measures of potentially related constructs of individual to response to difficult situations. Thus, the present study aimed to examine the relationship between the ATAS and the Japanese version of the Acceptance and Action Questionnaire (AAQ; Matsumoto and Okouchi 2012), an established scale for assessment of the therapeutic process of Acceptance and Commitment Therapy (ACT). We intended to reveal both positive and negative characteristic attitudes towards ambiguity by using the AAQ as a reference, given that response to ambiguity (ATAS) and such responses to negative stimuli (AAQ) may share some commonality in cognitive/emotional/behavioral expression.

Consequently, the main purpose of this study was to assess a new model of attitude towards ambiguity from a factor perspective, using a representative sample of the Japanese general population, together with a comparison of the ATAS (response to ambiguous situations) and the AAQ (response to unwanted situations).

## Method

### Participants

Initially, 1340 Japanese volunteers completed the questionnaires between November and December of 2013. The data of 321 participants were excluded due to past or present psychiatric illness, imprecise description of the participant’s background, or incomplete answers to the questionnaires. The final data set included 1019 participants (506 males and 513 females; mean age 34.1 years, *SD* = 12.7, age range 18–78 years and consisting of 102 teens, 336 twenties, 235 thirties, 218 forties, 93 fifties and 35 sixties or older), of which 687 were employed workers, 298 were vocational or university students and 34 were unemployed individuals (including homemakers). The respondents lived in Tokyo (100 participants), Osaka (113), and Okinawa (806) in Japan.

### Measures

Each participant completed the following two measures after initially providing demographic data: Age, sex, employment status (employed workers or unemployed individuals, including homemakers or students), and past/present psychiatric illness as an exclusion criterion.

#### Attitudes Towards Ambiguity Scale (ATAS)

The ATAS is a 26-item self-rating scale that assesses various attitudes towards ambiguous situations. Each item is scored using a six-point Likert scale (1 = *strongly disagree* to 6 = *strongly agree*). We used the original version of the ATAS (in the Japanese language), whose validity and reliability have been confirmed (Nishimura 2007).

With the permission of the original author (SN), the four authors of the present study (HE, MK, SS, and TK) translated the ATAS into English (see Appendix). However, the present investigation was conducted using the original version in Japanese.

A previous exploratory factor analysis of the ATAS in a university student sample (Nishimura 2007) revealed five subscales that described distinct attitudes towards ambiguity, i.e., enjoyment (7 items), anxiety (6 items), reception (5 items), control (5 items), and exclusion (3 items). The “enjoyment” factor indicates positive participation in ambiguous situations, with a stance of curiosity. The “anxiety” factor includes emotionally confused attitudes without active resolution when faced with ambiguous situations. The “reception” factor describes the attitude of accepting ambiguity, as it is. The “control” factor is the attitude of perceiving ambiguity as negative, and of coping with ambiguity rationally. The “exclusion” factor is associated with excluding ambiguity from reality to avoid feelings of discomfort (Nishimura 2007).

The ATAS has been evaluated in Japan as a multi-dimensional scale to assess attitudes towards ambiguity from the perspective of both positive (enjoyment and reception) and negative (anxiety, control and exclusion) reactions to ambiguous situations (Nishimura 2007).

#### Acceptance and Action Questionnaire (AAQ)

The AAQ is a self-rating scale that measures psychological flexibility or inflexibility, which is related to intolerant attitudes that drive one to alter the unwanted situation or its preceding contexts (Hayes et al. 1996). The AAQ can therefore be said to measure psychological flexibility in terms of experiential avoidance.

The original English version of the nine-item AAQ uses seven-point Likert scales to measure avoidance/acceptance of negative emotional experiences (Hayes et al. 2004). Thereafter, Bond and Bunce (2003) revised and expanded the original AAQ by developing a 16-item version, which consists of “Willingness” (7 items) and “Action” (9 items) subscales. The “Willingness” subscale assesses mindfulness and willingness to engage negative emotional experiences without changing sensations, thoughts, or emotions (Bond and Bunce 2003; Luoma et al. 2007). The “Action” subscale assesses the trait of taking positive action and “committing to reality” while accepting physiological and cognitive responses elicited during the situation and being free from acting in accordance with unhelpful rules and verbal events, even when faced with difficult and disappointing situations (Bach and Moran 2008).

We used the Japanese version of the AAQ (Matsumoto and Okouchi 2012), which slightly modifies the scales of Bond and Bunce (2003), and whose validity and reliability have been confirmed (Matsumoto and Okouchi 2012). The AAQ was used as a reference against which the ATAS components were assessed, given that both AAQ and ATAS appear to operationalize similar concepts, encompassing cognitions and coping patterns towards undesired situations.

### Ethics

This study was approved by the Ethical Review Board for Epidemiologic Study of the University of the Ryukyus. Participants reviewed documents that explained the purpose of the study, emphasized that participation was voluntary, provided details regarding protection of personal information, noted the right to withdraw from the study, highlighted possible personal benefits, and explained the expected contribution of the study to society. All participants provided their data anonymously. Only coded and grouped data were used for analyses.

### Statistical Analyses

Exploratory factor analysis was initially tried to extract the dimensional structure of the ATAS (Table 1), using a larger sample with a wider age range than previously reported (Nishimura 2007). Confirmative factor analysis was also conducted, based on the original five-factor model proposed by a previous study (Nishimura 2007). Then, we compared the goodness-of-fit indexes between newly extracted factor model and the 5 factor model (Table 2). The effects of age and sex on the ATAS and AAQ subscales were also analyzed using Pearson correlations and the point-biserial correlations (Table 3). Pearson correlations were used to assess the relationships among the ATAS subscales (Table 4). Relationships between the ATAS and AAQ subscales were explored using Pearson correlations, after controlling for age and sex (Table 5). A two-tailed *p-*value of less than .05 was regarded as statistically significant. SPSS 19.0.1 for Windows and AMOS 19.0 (IBM Japan Inc., Tokyo, Japan) were used for statistical analyses.

## Results

### Exploratory Factor Analysis of the ATAS

The subjects of the previous ATAS study (Nishimura 2007) were all university students (mean age ± *SD*: 19.7 ± 1.7 years), which did not reflect general population. Thus, the present study aimed to explore the factor structure of ATAS again in large and non-biased representative samples of general population. Thereby, we initially performed exploratory factor analysis.

ATAS score distributions did not deviate from normality, based on checking for ceiling and floor effects. We then applied exploratory factor analysis using an unweighted least-squares method with Promax rotation. We adopted a 4-factor model of the ATAS, based on scree test and eigenvalue interpretation/reduction (5.16, 3.67, 2.40, 1.14, 1.05, and .98). We then conducted another factor analysis via the least-squares method with Promax rotation, based on the assumption of a 4-factor model of 25 ATAS items (factor loading ≥ .38), after excluding item #20 due to its very low factor loadings (see Table 1).

Four distinctive attitudes towards ambiguity were extracted with good internal consistency (Table 1), namely Enjoyment (12 items, Cronbach’s *α* = .83), Anxiety (6 items, α = .75), Exclusion (4 items, α = .75), and Noninterference (3 items, α = .65).

**Table 1:**
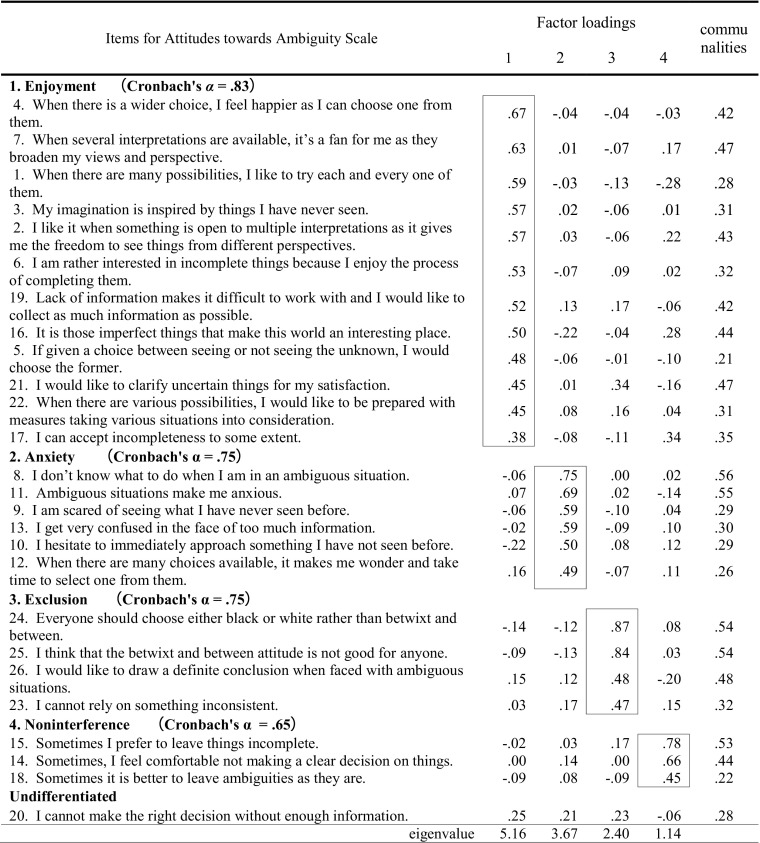
Exploratory factor analysis of the 26 items for Attitudes towards Ambiguity Scale, with Promax rotation

### Confirmatory Factor Analysis of the ATAS

Confirmatory factor analysis by maximum likelihood estimation method was also conducted using larger number of general subjects with wider age range to confirm the original five-factor model by a previous exploratory factor analysis of rather specific samples of university students (Nishimura 2007). Multiple goodness of fit indices was evaluated using Chi-square test, the goodness-of-fit index (GFI: Jöreskog and Sörbom 1986), adjusted goodness-of-fit index, (AGFI: Jöreskog and Sörbom 1986), the comparative fit index (CFI: Bentler 1990) and the root-mean-square error of approximation (RMSEA: Steiger and Lind 1980). The results were acceptable, but were not necessarily the best fit (*χ2* = 1754.75, *df* = 289, GFI = 0.868, AGFI = 0.84, CFI = 0,806, RMSEA =0.071 [CI: 0.067–0.074] ), which were comparable to the present four-factor model (*χ2* = 1983.17, *df* = 269, GFI = 0.844, AGFI = 0.811, CFI = 0,762, RMSEA =0.079 [CI: 0.076–0.082] ), as shown in Table 2.

**Table 2:** Goodness of fit indices of confirmatory factor analysis

	*χ* ^*2*^	*df*	GFI	AGFI	CFI	RMSEA (90%CI)
Four-factor model	1983.17	269	0.844	0.811	0.762	0.079
						(0.076, 0.082)
Five-factor model	1754.75	289	0.868	0.840	0.806	0.071
						(0.067, 0.074)

*GFI* Goodness-of-fit index, *AGFI* Adjusted goodness-of-fit index, *CFI* Comparative fit index, *RMSEA* Root mean square error of approximation

As for significant differences in these indices between both models, it has been suggested that discrepancies more than 0.01 in CFI and 0.015 in RMSEA are necessary (Chen 2007). With regard to this, significant superiority of either model to the other was not applicable to the latter criteria for RMSEA (Table 2). Chen (2007) also warned that a model with a larger sample size can be accidentally rejected when all fit indices were affected by decreased standard deviations according to increased sample sizes. Considering much larger number of representative samples with general population in the present study, we adopted the four-factor model based on interpretability from the present exploratory factor analysis. 

### Effects of Back Grounds (Locality, Employment Status, Age and Sex) ATAS and AAQ Subscales

There were no significant effects of respondent location (Tokyo/Osaka/Okinawa) or employment status (employed/unemployed) on any of the ATAS (Enjoyment, Anxiety, Exclusion, and Noninterference) or AAQ subscales (Willingness and Action). Thus, the 1019 subjects were regarded as one group for statistical analyses, irrespective of their employment status and locality.

Among the ATAS subscales, only Anxiety subscale scores were significantly greater for younger (*r* = −.26, *p* < .001) and female participants (*r* = .20, *p* < .001); the other three subscales were not significantly affected by age or sex (Table 3).

**Table 3:** Correlations of age and sex Acceptance and Action Questionnaire (AAQ) and Attitudes towards Ambiguity Scale (ATAS) subscales

	AAQ		ATAS			
	Willingness	Action	Enjoyment	Anxiety	Exclusion	Noninterference
Age	.11**	.17***	-.02	-.26***	-.06	.02
95%CI	(.05, .17)	(.11, .23)	(−.08, .04)	(−.32, −.21)	(−.12, .01)	(−.04, .08)
Sex	-.07*	-.02	-.06	.20***	.01	.01
95%CI	(−.13, −.01)	(−.08, .04)	(−.12, .00)	(.14, .26)	(−.05, .07)	(−.06, .07)

* *p* < .05, ** *p* < .01, *** *p* < .001

For the AAQ subscales, a positive but weak correlation was found between age and Willingness (*r* = .11, *p* < .01) as well as Action (*r* = .17, *p* < .001), whereas sex had a negligible effect on the AAQ subscales (Table 3). 

### Correlations among the ATAS Subscales

Significant correlations were found between all ATAS subscales (Table 4). Weak correlations were observed between Enjoyment and Exclusion (*r* = .26, *p* < .001) and Noninterference (*r* = .22, *p* < .001), and a moderate correlation was found between Anxiety and Exclusion (*r* = .37, *p* < .001).

**Table 4:** Correlations among four Attitudes towards Ambiguity Scale (ATAS) factors

	Mean (*SD*)	1	2	3
Scale ATAS				
1. Enjoyment	51.79 (8.49)			
95%CI				
2. Anxiety	21.86 (5.55)	.07*		
95%CI		(.01, .13)		
3. Exclusion	15.24 (3.90)	.26***	.37***	
95%CI		(.20, .32)	(.32, .43)	
4. Noninterference	10.41 (3.06)	.22***	.10**	-.19***
95%CI		(.17, .28)	(.04, .16)	(−.25, −.13)

* *p* < .05, ** *p* < .01, *** *p* < .001

### Correlations among ATAS and AAQ Subscales

A partial correlation analysis was conducted to examine relationships among the ATAS and AAQ subscales after controlling for age and sex (Table 5). The ATAS Anxiety subscale was negatively correlated with the AAQ Willingness subscale (*r* = −.39, *p* < .001), whereas ATAS Enjoyment was positively correlated with AAQ Action (*r* = .40, *p* < .001). Weak correlations were found between ATAS Exclusion and AAQ Willingness (*r* = −.20, *p* < .001) and Action (*r* = .16, *p* < .001).

**Table 5:** Correlations between Acceptance and Action Questionnaire (AAQ) and Attitudes towards Ambiguity Scale (ATAS) subscales controlling for age and sex

		ATAS			
	Mean (*SD*)	Enjoyment	Anxiety	Exclusion	Noninterference
Willingness	20.80 (5.23)	.05	-.39***	-.20***	-.04
95%CI		(−.01, .11)	(−.44, −.33)	(−.26, −.14)	(−.10, .02)
Action	23.25 (4.82)	.40***	-.09**	.16***	.02
95%CI		(.35, .45)	(−.15, −.03)	(.10, .22)	(−.04, .08)

** *p* < .01, *** *p* < .001

## Discussion

### Four-Factor Model of the ATAS in a General Population

Factor analysis of the 26 items of the ATAS, using the responses of 437 Japanese university students, initially revealed a five-factor model (Nishimura 2007). In the present study, reexamination of the multi-dimensional structure of the ATAS using a larger sample (*N* = 1019) with an even sex distribution and a broader age range, a sample more representative of the general population in Japan, clearly demonstrated a four-factor model based on 25 items (item #20 was excluded due to low factor loadings, as shown in Table 1). The original classification of the ATAS subscales (see Appendix) by Nishimura (2007) was enjoyment (7 items: #1–7), anxiety (6 items: #8–13), reception (5 items: #14–18), control (5 items: #19–23), and exclusion (3 items: #24–26). In the present study, the items were rearranged according to the obtained four-factor structure (see Table 1): Enjoyment (12 items: #1–7, 16, 17, 19, 21 and 22), Anxiety (6 items: #8–13), Exclusion (4 items: #23–26), and Noninterference (3 items: #14, 15 and 18).

The Enjoyment subscale (12 items) derived here completely subsumes the seven items of the original Enjoyment subscale and further includes two from the original reception subscale (#16 and 17), and three from the original control subscale (#19, 21, and 22). Nishimura (2007) originally conceptualized the enjoyment element as a positive emotion (i.e., perceiving ambiguity as attractive and pleasurable). In contrast, the new Enjoyment subscale derived here may additionally encompass an enjoyable acceptance of incompleteness, but also a readiness to clarify ambiguous situations.

The six-item Anxiety subscale obtained here is identical to the original six-item anxiety subscale. This subscale was originally defined as “emotional confusion”, or namely, being anxious in unfamiliar/complicated situations and feeling difficulty with coping with such situations (Nishimura 2007).

The present four-item Exclusion subscale consists of the original exclusion subscale (3 items) with the addition of one item (#23) from the original control subscale of Nishimura (2007). Thus, the new Exclusion component may also include rejection of inconsistency, in addition to decision-making based on dichotomous thinking, mainly from a behavioral perspective.

The Noninterference subscale is newly developed in the present study. Its three items (#14, 15, and 18) were extracted from the original five-item reception subscale. The new subscale may simply imply neutral passive attitudes, such as taking no action and leaving the ambiguous situation as it is. These attitudes do not belong to either enjoyable acceptance (positive) or anxious avoidance (negative).

Confirmative factor analysis failed to show significant differences in goodness in fit indices between the new four-factor model from the present exploratory factor analysis and the original five-factor model in a previous study (Nishimura 2007). The weak reproducibility of the five-factor model can be at least partly explained by the differences in subjects (a large number of general population samples in the present study versus a small number of young university students in the preceding study). Also in the study by Nishimura (2007), re-test reliability revealed relatively weak reproducibility in reception (.59) and control (.64) compared with steady components like enjoyment (.73), anxiety (.76) and exclusion (.72). The latter 3 elements (enjoyment, anxiety and exclusion) survived in the present study. The 3 items of the newly defined “noninterference” factor in the present study came out of the original 5-item “control” factor in our previous study. However, the original “reception factor disappeared and absorbed in the new “enjoyment” factor in the present study.

Therefore, the new 4-factor model in the present study shares common factor structure with slight modification, in comparison with the original 5-factor model in our previous study, Abovementioned minor changes are probably due to weak cohesiveness of the original “control” and “reception” factors and large difference in age distribution in the two studies. Although the original 5-factor model was integrated into a new 4-factor model, core structure was regarded to be unchanged and reproducible.

### Proposal for a 2 × 2 Dimensional Structure of the ATAS

Nishimura’s (2007) five-factor model of the ATAS consists of positive (enjoyment and reception) and negative attitudes (anxiety, control, and exclusion). However, the present study revealed a four-factor model of the ATAS, wherein items of the original reception subscale were integrated into Enjoyment and Noninterference. Likewise, the majority of the original control subscale items were subsumed into the Enjoyment subscale, with the remainder becoming components of the Exclusion subscale.

The Enjoyment and Anxiety subscales appear to capture contextual cognitive/emotional responses to ambiguity, while the Exclusion and Noninterference subscales assess more behavior-oriented responses to the ambiguity. Furthermore, the Enjoyment and Exclusion subscales appear to describe active/dynamic attitudes towards ambiguity, while Anxiety and Noninterference are likely to represent passive/static attitudes. Therefore, the four-factor model of the ATAS can be hypothesized as a 2 × 2 dimensional structure, i.e., the psychological basis of attitudes (cognitive/emotional vs behavioral) versus the dynamics of the attitudes (active/dynamic vs passive/static). Accordingly, the four ATAS factors may be summarized as Enjoyment as active cognition/emotion, Anxiety as passive cognition/emotion, Exclusion as an active behavior, and Noninterference as a passive behavior.

### Comparison with a recently Proposed Multi-Dimensional Model of Attitudes Towards Ambiguity

A recent study by Lauriola et al. (2015) developed a 30-item Multidimensional Attitude Towards Ambiguity Scale, which consists of three factors: affective (Discomfort with Ambiguity: DA), cognitive (Moral Absolutism/Splitting: MA/SPLT), and epistemic (Need for Complexity and Novelty: NC). This scale has some structural similarities with the original ATAS: DA and NC are almost identical to the Anxiety and Enjoyment components of the ATAS, respectively.

We propose that the ATAS (Nishimura 2007) may have advantages over the scale of Lauriola et al. (2015), because the former comprehensively assesses both cognitive/affective (Anxiety and Enjoyment) and behavioral components (Exclusion and Noninterference). Unfortunately, the MA/SPLT factor of Lauriola et al. (2015) only encompasses the preference dichotomy at the cognitive level, which may not directly lead to a decision to take action at the behavioral level. In addition, the same authors have only focused on negative aspects of the attitudes whereas our model covers broader concept, including active behavioral elements of attitudes towards ambiguity and additionally estimates positive aspects of the attitudes. Additionally, Lauriola et al. (2015) considered two different and specific populations, i.e., Italian undergraduate students and US residents, with data collected through the Amazon Mechanical Turk online service. As such, this sample may not be representative of the general population. Therefore, the Lauriola et al. factor model should be carefully interpreted and reexamined in a larger population, using an unbiased sampling method that encompasses a wider age range.

### Correlations and Influential Factors among the ATAS Subscales

Among the ATAS subscales, Enjoyment was significantly correlated with both Exclusion and Noninterference, while Anxiety was strongly correlated with Exclusion (Table 4). It appears that one who enjoys ambiguous situations takes two distinct attitudes, i.e., excluding ambiguity from active resolution, or not interfering with ambiguity due to good tolerance of this experience. In contrast, one who tends to be anxious about ambiguity may exclude the ambiguity due to intolerance, leading to lowered acceptance of his/her feelings and of the reality of the situation.

Age and sex at least partly affected the ATAS Anxiety subscale (Table 3). The negative correlation between age and the Anxiety subscale demonstrates that younger subjects, with less life experience, tend to become more anxious and confused when faced with ambiguous situations. Meanwhile, it appears that females are more susceptible to ambiguous situations and tend to show more anxiety, which is consistent with a previous finding that females are more likely to have an anxious temperament than males (Koda and Kondo 2010).

### Association between the ATAS and AAQ

The well-established AAQ consists of two factors: Willingness and Action (Bond and Bunce 2003). Willingness is the concept of being willing to fully experience feelings as they are, even though such experiences may be unpleasant or painful (Walser and Westrup 2007). Twohig et al. (2006) mentioned that increased willingness helps to reduce the “experimental avoidance” noted by Hayes et al. (1996). In addition, it is desirable to engage in effective behavior, even when faced with unwanted internal events (Hayes et al. 1996).

We investigated the relationship between the AAQ and the ATAS. The main difference between these two scales is the specific nature of the response target, in that the given situations are unpleasant in the AAQ, whereas they are unpredictable in the ATAS. Thus, the AAQ solely deals with responses to negative stimuli, whereas the ATAS deals with inter-individual variation in responses to ambiguous situations. Nevertheless, these assessments may overlap to some extent. The AAQ and aspects of the ATAS may similarly measure individual capability for acceptance of the present situation as it is. Moreover, both scales deal with maladaptive aspects of individual attitudes towards surrounding situations (i.e., negative affect or responses). Therefore, it may be of great interest for mental health professionals to understand the differences and similarities between the AAQ and the ATAS.

Based on the negative correlations between ATAS Anxiety/Exclusion and AAQ Willingness (Table 5), experiencing ambiguity without anxious attitudes or exclusion-type behavior may lead to acceptance of unpleasant internal states without avoiding or trying to control them. This result may be partly explained by previous findings suggesting an inverse relationship between anxiety and willingness (Bendayan et al. 2012; Bluett et al. 2014; Hayes et al. 2004) and a positive relationship between anxiety and experiential avoidance (Tull and Gratz 2008).

There was also a positive correlation between ATAS Enjoyment and AAQ Action (Table 5). This suggests that not only emotional aspects (enjoyable acceptance of the ambiguity) but also behavior-oriented aspects (readiness to clarify ambiguous situations) may lead to positive and effective commitment during unpleasant internal experiences. Specifically, the enjoyment of ambiguity is not restricted to inner feelings of pleasure (self-satisfaction) but also promotes positive adjustment to difficult situations. Although the assessment targets differ for the ATAS and AAQ, as described above (ambiguous situations for the ATAS and unpleasant experiences for the AAQ), the scales seem to measure related constructs and share common channels for assessments of cognitive/emotional/behavioral patterns in individuals facing difficulties.

### Future Perspectives

Assessment of multi-dimensional attitudes towards ambiguity using the ATAS may have several clinical implications. First, the relationship between personality traits and attitudes towards ambiguity is of great interest, since personality has been regarded as the most important factor that affects individual cognitive, emotional, and behavioral patterns. Such psychological connectivity may provide a new perspective on individual psychopathology.

Second, profiles of attitudes towards ambiguity may differ across psychiatric conditions (e.g., anxiety, obsessive-compulsive disorders, depression, and neurodevelopmental disorders). Thus, clarification of the attitudes present in each psychiatric condition may enhance our understanding of psychopathology, and thus suggest more effective strategies for psychotherapeutic intervention.

Third, there may be transcultural effects on attitudes towards ambiguity among countries with different religions, cultures, and societies. Since individual cognition, emotion, and behavior are at least partly culture-bound, differences in ATAS profiles may provide a new perspective on the national character of different countries. We encourage English-speaking researchers to examine the reliability and validity of the English translation (Appendix).

### Conclusion

Factor analysis extracted four components from the ATAS, namely Enjoyment, Anxiety, Exclusion, and Noninterference as attitudes towards ambiguous situations. Among the ATAS subscales, positive correlations were found between Enjoyment and the Exclusion and Noninterference subscales, and between Anxiety and Exclusion. The Anxiety subscale of the ATAS was negatively correlated with the Willingness subscale of the AAQ, while ATAS Enjoyment was positively correlated with AAQ Action.

It is thus suggested that one who enjoys ambiguous situations can adopt two distinct attitudes: Excluding ambiguity from active resolution, or not interfering with ambiguity due to good tolerance of this experience, which can lead to positive and flexible commitments in life. In contrast, one who tends to be anxious about ambiguity may be characterized by exclusion-based attitudes due to intolerance of ambiguity, leading to lowered acceptance of their feelings and of the reality of circumstances, especially in younger females.

### Limitations

The present study has some limitations. First, we did not compare the ATAS with other related scales that assess tolerance of ambiguity, although a previous study using the original Japanese version of the ATAS (Nishimura 2007) had already confirmed a close relationship between the ATAS and the modified Japanese version (Masuda 1998) of the Measurement of Ambiguity Tolerance (MAT-50; Norton 1975). Second, we used the first version of the AAQ, given that the latest version (AAQ-II; Bond et al. 2011) has yet to be translated into Japanese. Third, we await future research investigating the reliability and validity of the English version of the ATAS, as presented in Appendix.

## Figures and Tables

**Table 6:**
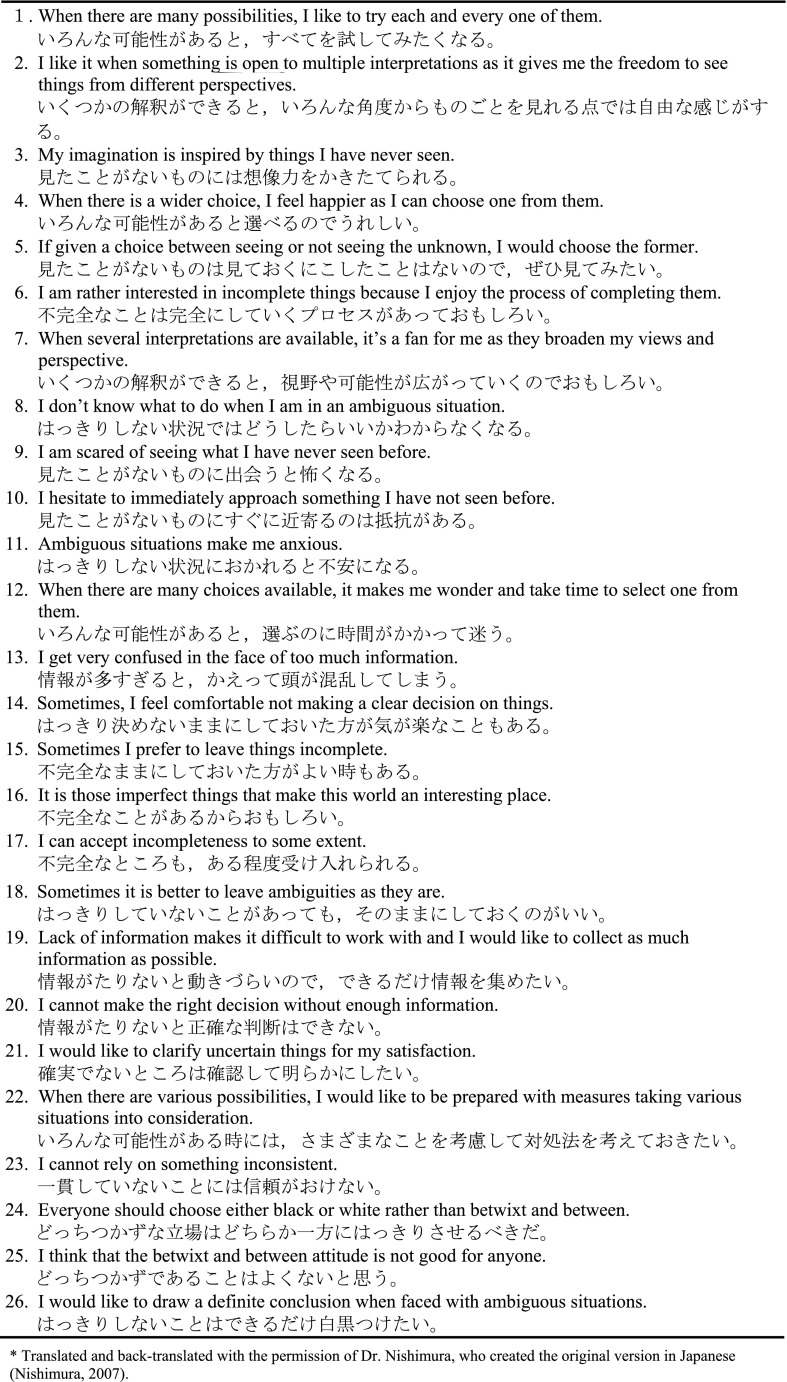
English translation of the original Japanese version of the “Attitudes towards Ambiguity Scale”

## Appendix

English translation of the original Japanese version of the “Attitudes towards Ambiguity Scale”
